# Abdominal Wall Reinforcement Using OviTex after Deep Inferior Epigastric Perforator Flap

**DOI:** 10.1055/a-2555-2348

**Published:** 2025-04-01

**Authors:** Alec S. McCranie, Caitlin Blades, Steven Dawson, Jose A. Foppiani, Taylor Allenby, Julian Winocour, Justin Cohen, David Mathes, Christodoulos Kaoutzanis

**Affiliations:** 1 Division of Plastic and Reconstructive Surgery, Department of Surgery, University of Colorado Anschutz Medical Campus, Aurora, Colorado; 2 Department of Plastic and Reconstructive Surgery, Beth Isreal Deaconess Medical Center, Boston, Massachusetts

**Keywords:** deep inferior epigastric perforator flap, mesh, breast reconstruction, hernia, bulge

## Abstract

**Background:**

Abdominal wall bulges and hernias are not uncommon complications following deep inferior epigastric perforator (DIEP) flap harvest. Abdominal wall reinforcement using synthetic meshes has been found to decrease bulges by up to 70%; however, such meshes can be associated with other issues such as seromas and infections. Reinforced tissue matrix (RTM) mesh can be used for abdominal wall reinforcement due to its ability to recruit fibroblasts and provide a scaffold for cellular proliferation. There is no literature on the use of OviTex mesh for abdominal wall reinforcement following DIEP flap harvest. Therefore, this study aimed to evaluate the efficacy and safety of its use in this setting.

**Methods:**

A retrospective review was performed on patients undergoing DIEP flap harvest between January 2020 and June 2023. Patients who had completed at least 12 months of follow-up visits were included. Descriptive, univariate, and multiple logistic regression analyses were completed.

**Results:**

A total of 199 patients were included. The mean age at the time of surgery was 51.1 ± 10.0 years and the mean body mass index (BMI) was 30.2 ± 5.9 kg/m ^2^ . Abdominal wall reinforcement was completed in 85 (42.7%) patients. Patients who had OviTex placed developed fewer bulges compared to the non-mesh cohort (0% vs. 5.3%, *p* = 0.04). Furthermore, OviTex mesh did not increase adverse events and was not significantly different in seroma/hematoma rates when compared to the non-mesh cohort (10.6% vs. 5.3%, *p* = 0.26).

**Conclusion:**

This study demonstrates that OviTex mesh is safe and efficacious in reducing the rate of bulges following DIEP flap harvest without increasing other complications.

Deep inferior epigastric perforator (DIEP) free flaps have been referred to as the “gold standard” treatment for autologous breast reconstruction. 1 However, abdominal wall bulges and hernias are known potential complications following DIEP flap harvest. 1 2 3 Bulges, in particular, are a difficult problem to address postoperatively as there is no identifiable facial defect that can be fixed and the cause of developing abdominal bulging after DIEP flap harvest is not fully understood. 4 Bulges can occur between 2.3% and 33% and hernias can occur between 0% and 7% after DIEP flap harvest. 5 6 7 8 9 10 When used to reinforce the abdominal wall following DIEP flap harvest, synthetic meshes such as polypropylene mesh have been successful in decreasing bulges and hernias by up to 70%. 1 11 12 However, such meshes have an association with an increased rate of seroma development, infection, and chronic inflammation, which has been attributed to the synthetic material. 13 14 15 When severe, these complications may require additional surgery for mesh removal. 16

An alternative to using a synthetic mesh is a mostly absorbable reinforced tissue matrix (RTM), such as OviTex. This matrix has the advantage of recruiting fibroblasts, providing a scaffold for cell proliferation, and minimizing any foreign body response. 17 There is no current literature supporting the use of OviTex for abdominal wall reinforcement following DIEP flap harvest. Therefore, the aim of our study was to evaluate the safety and efficacy of OviTex mesh for abdominal wall reinforcement following DIEP flap harvest for autologous breast reconstruction.

## Methods

### Study Design and Setting

A retrospective cohort study was performed on patients who underwent unilateral or bilateral DIEP flap harvest for autologous breast reconstruction at a large academic institution between January 2020 and June 2023. The Strengthening the Reporting of Observational Studies in Epidemiology (STROBE) guidelines were followed. 18 The study was approved by the Institutional Review Board (IRB) before any study-related activities (IRB# 23-0325).

### Participants

Inclusion criteria for the study involved female patients 18 years or older who underwent unilateral or bilateral DIEP flap breast reconstruction at a large institution between January 2020 and June 2023. All patients that were reviewed met the study's inclusion criteria. All patients had a minimum of a 12-month follow-up period.

### Mesh use

**Video 1** Intraoperative technique for securing the mesh in a sublay position.


**Fig. 1:**
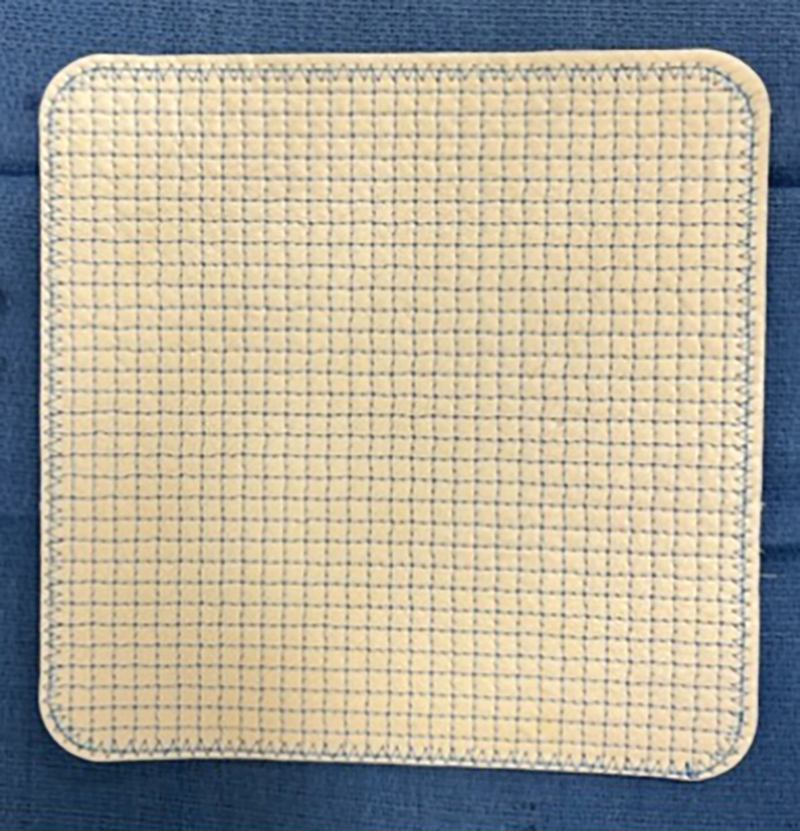
Intraoperative photograph of OviTex mesh.

**Fig. 2:**
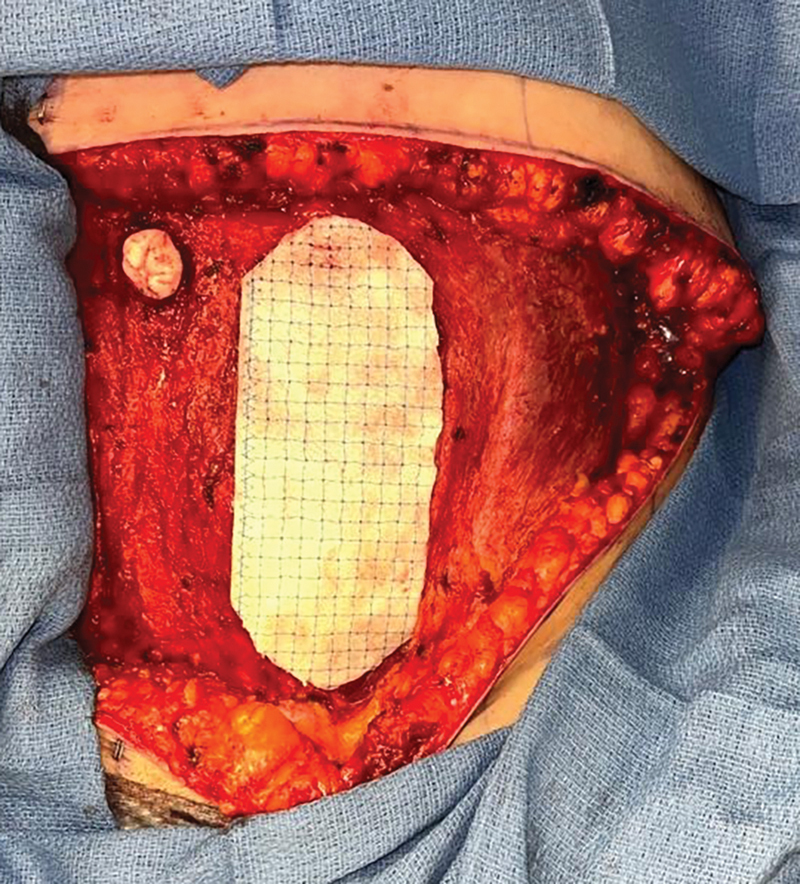
Intraoperative photograph of OviTex mesh cut into an oval shape.

**Fig. 3:**
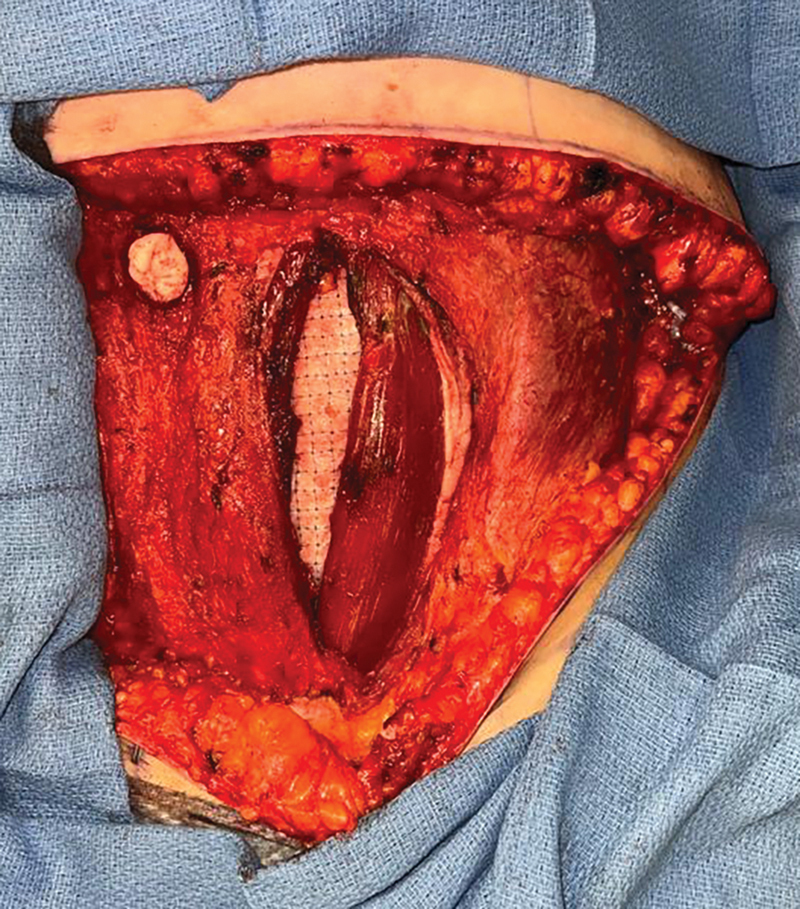
Intraoperative photograph of OviTex mesh placed in the retro-rectus space in a sublay position.

**Fig. 4:**
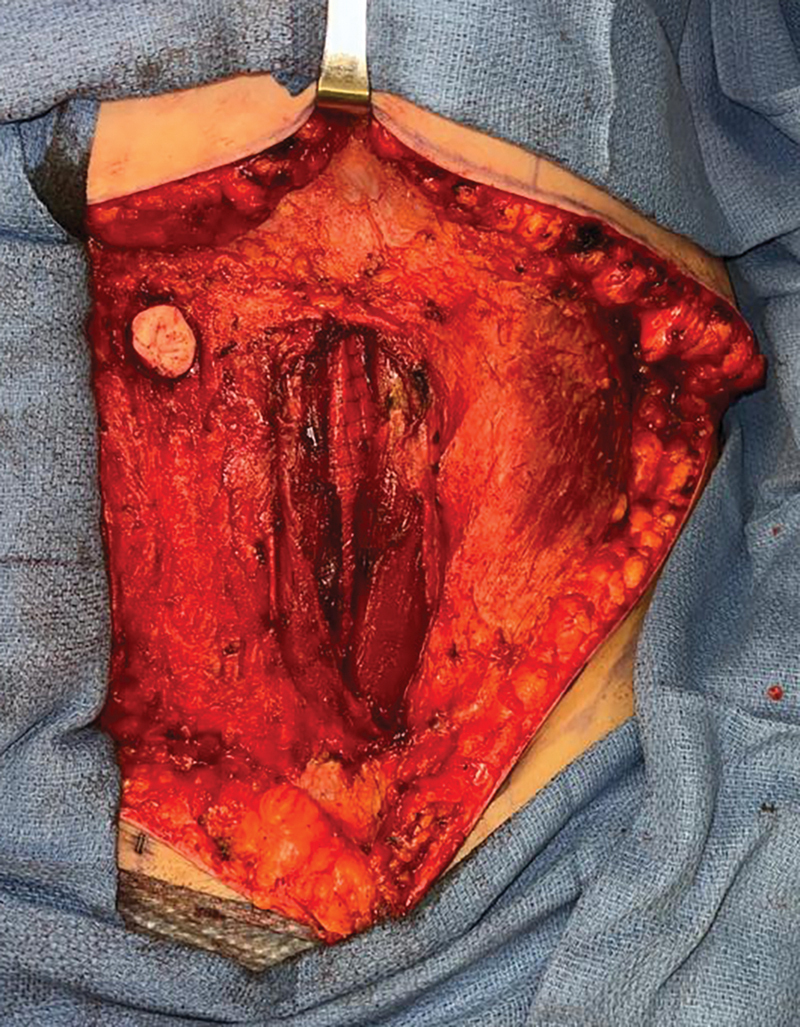
Intraoperative photograph of OviTex mesh in a sublay position secured to the anterior rectus sheath.

The RTM mesh that was evaluated in this study was the OviTex Core RTM (OviTex Core; TELA Bio, inc.). OviTex mesh is a fenestrated xenograft made of four layers of ovine extracellular matrix. 15 The matrix is reinforced by permanent polypropylene monofilament which is woven throughout the matrix in a diamond-shaped pattern ( Fig. 1 ). After the harvest of the DIEP flap, the retro-rectus space is developed as needed to accommodate the placement of the OviTex mesh by gentle manual dissection or bovie electrocautery. Typically, not much of an additional dissection of the retro-rectus space is needed. Of note, if dissection is necessary, all the nerves are preserved because the dissection is done bluntly and carefully with a digit. Next, the OviTex mesh is prepared as per the manufacturer's guidelines and cut with scissors to the size of the defect, usually in an oval shape ( Fig. 2 ). It is then placed in the retro-rectus space, as a sublay ( Fig. 3 ), and secured to the anterior rectus sheath with six to eight 2–0 Vicryl sutures that are passed through the rectus abdominis muscle in the majority of cases in a horizontal mattress fashion ( Video 1 ). The mesh is secured with adequate stretch to prevent laxity of the mesh after closure of the anterior abdominal wall. Once the mesh is in a good position ( Fig. 4 ), the anterior rectus sheath is closed primarily in two layers. First, it is approximated with buried figure-of-eight permanent or long absorbing sutures followed by a running long absorbing suture to reinforce the closure. All patients regardless of mesh placement received antibiotics prior to surgery and for 24 hours postoperatively. Additionally, the surgeons included in this study had experience performing DIEP flap harvests with mesh placement for the donor side prior to the start of this study.

### Data Collection

Electronic medical records for each patient were manually reviewed. Patient demographics and characteristics including age, race, ethnicity, insurance status, body mass index (BMI), smoking status, alcohol status, and medical comorbidities included in the Charleston Comorbidity Index (CCI) and American Society of Anesthesiologists physical status classification (ASA) were collected. The patient's cancer stage and oncological treatment were also collected. Operative details included mastectomy side and type, reconstruction timing, perforators used, operating room (OR) time, and use of mesh. Abdominal complications were reviewed and consisted of wound dehiscence, surgical site infection (SSI), seroma, hematoma, bulge, hernia, emergency department (ED) visits related to an abdominal complication, and reoperations related to an abdominal complication. A bulge was defined as an intact but thinned abdominal wall on a computed tomography scan resulting in bulging and a hernia as a defect in the abdominal wall fascia. The primary outcome of this study was the efficacy of OviTex mesh in reducing abdominal wall-related complications.

### Statistical Analysis

Descriptive statistics were used to summarize characteristics identified during the chart review process. Patient demographics and clinical characteristics were tested using Pearson's chi-square tests. Fisher's exact tests were used when 25% or more table cells had expected counts less than 5. A multiple logistic regression was used to evaluate if preoperative and operative characteristics are associated with developing a bulge. For all statistical tests, a value of *p* < 0.05 was considered statistically significant. Statistical analysis was performed using R Software, version 4.3.0, and R Studio, version 2023.03.1 (I 2022 Posit Software, Public Benefit Corporation).

## Results

A total of 199 patients who underwent autologous breast reconstruction with DIEP flaps were identified during the study period. The mean age of the patients at the time of surgery was 51.1 ± 10.0 years and the mean BMI was 30.2 ± 5.9 kg/m ^2^ . The mean follow-up period was 27.2 months. Patient comorbidities are summarized in Table 1 . DIEP flap surgery was performed in 138 (69.3%) patients with stage I to III breast cancer, 23 (11.6%) with ductal carcinoma in situ, 5 (2.5%) with metastatic disease, 27 (13.6%) for prophylactic reasons, and 6 (3.0%) who did not have a documented cancer stage. These six patients presented from an outside institution. A unilateral mastectomy was performed in 59 (29.6%) patients, while 139 (69.8%) patients underwent a bilateral mastectomy. A skin-sparing mastectomy was performed in 152 (76.4%) patients, nipple-sparing mastectomy in 42 (21.1%), and modified radical mastectomy in 4 (2.0%). There were 54 (27.1%) patients who had immediate reconstruction and 144 (72.4%) who underwent delayed reconstruction. The overall mean OR time was 537.3 ± 113.3 minutes. The mean OR time was not significantly different between patients who had mesh placed, 533.1 ± 107.8 minutes, and those who did not have mesh, 540.4 ± 117.5 minutes ( *p* = 0.65).

**Table 1:** Patient, cancer, and reconstruction characteristics of the OviTex mesh and non-mesh cohorts

Parameter	OviTex mesh ( *N* = 85)	No mesh ( *N* = 114)	*p* -Value
Age, years (mean ± SD)	51.9 ± 10.4	50.5 ± 9.6	0.32
BMI, kg/m ^2^ (mean ± SD)	29.9 ± 5.9	30.4 ± 6.0	0.54
BMI greater than 30 kg/m ^2^ , *N* (%)	38 (44.7)	59 (51.8)	0.40
CCI (mean ± SD)	2.5 ± 1.7	2.6 ±1.5	0.76
Follow-up, days (mean ± SD)	629.4 ± 186.7	951.7 ± 260.5	<0.01
Race, *N* (%)			0.50
American Indian or Alaska Native	0 (0)	1 (0.88)	
Asian	2 (2.4)	3 (2.6)	
Black or African American	6 (7.1)	4 (3.5)	
Native Hawaiian or Other Pacific Islander	1 (1.2)	0 (0)	
White	69 (81.2)	99 (86.8)	
More than one race	7 (8.2)	5 (4.4)	
Unknown	0 (0)	1 (0.88)	
Ethnicity, *N* (%)			0.24
Hispanic	12 (14.1)	9 (7.9)	
Non-Hispanic	73 (85.9)	105 (92.1)	
Insurance status, *N* (%)			0.54
Medicare	9 (10.6)	8 (7.0)	
Medicaid	13 (15.3)	13 (11.4)	
Private	59 (69.4)	89 (78.1)	
Self-pay	0 (0)	1 (0.88)	
Military insurance	4 (4.7)	3 (2.6)	
ASA, *N* (%)			0.17
1	1 (1.2)	1 (0.88)	
2	53 (62.4)	84 (73.7)	
3	31 (36.5)	29 (25.4)	
Tobacco use, *N* (%)	2 (2.4)	1 (0.88)	0.78
Diabetes, *N* (%)	5 (5.9)	7 (6.1)	1.0
Chronic lung disease, *N* (%)	20 (23.5)	25 (21.9)	0.92
Cancer stage, *N* (%)			0.78
Stage 0 (DCIS)	10 (11.8)	13 (11.4)	
Stage IA	12 (14.1)	25 (21.9)	
Stage IB	8 (9.4)	6 (5.3)	
Stage IIA	17 (20.0)	16 (14.0)	
Stage IIB	13 (15.3)	12 (10.5)	
Stage IIIA	5 (5.9)	7 (6.1)	
Stage IIIB	3 (3.5)	7 (6.1)	
Stage IIIC	3 (3.5)	4 (3.5)	
Stage IV	2 (2.4)	3 (2.6)	
Prophylactic	10 (11.8)	17 (14.9)	
Mastectomy side, *N* (%)			1.0
Unilateral	25 (29.4)	34 (29.8)	
Bilateral	59 (69.4)	80 (70.2)	
Mastectomy type, *N* (%)			0.57
Skin-sparing	67 (78.8)	85 (74.6)	
Nipple-sparing	15 (17.6)	27 (23.7)	
Radical	2 (2.4)	2 (1.8)	
Oncology treatment, *N* (%)			
Neoadjuvant chemotherapy	27 (31.8)	50 (43.9)	0.11
Neoadjuvant radiation	1 (1.2)	2 (1.8)	1.0
Adjuvant chemotherapy	30 (35.3)	28 (24.6)	0.14
Adjuvant radiation	43 (50.6)	53 (46.5)	0.67
Hormone therapy	46 (54.1)	62 (54.4)	1.0
Prophylactic	9 (10.6)	17 (14.9)	0.50
None	9 (10.6)	12 (10.5)	1.0
Reconstruction timing, *N* (%)			0.24
Immediate	19 (22.4)	35 (30.7)	
Delayed	66 (77.6)	78 (68.4)	

Abbreviations: ASA, American Society of Anesthesiologists physical status classification; BMI, body mass index; CCI, Charleston Comorbidity Index; DCIS, ductal carcinoma in situ; SD, standard deviation.

Of the 199 patients included in the study, abdominal wall reinforcement with OviTex was used in 85 (42.7%) patients. The patient characteristics of the OviTex mesh and non-mesh cohorts are summarized in Table 1 . The perforators that were used for both the left-sided flap ( *p* = 0.25) and the right-sided flap ( *p* = 0.06) were similar between the no-mesh and Ovitex-mesh cohorts. There were more patients with two rows of perforators used in the Ovitex-mesh cohort compared to the no-mesh cohort (35.3% vs. 20.9%, *p* = 0.03). Overall, six (3.0%) patients developed a bulge, and two (1.0%) patients developed a hernia postoperatively. The OviTex cohort had no bulges when compared to six (5.3%) bulges in the non-mesh cohort which was significant ( *p* = 0.04). The OviTex cohort had no hernias when compared to two (1.8%) in the non-mesh cohort, but the difference was not statistically significant ( *p* = 0.51). The use of one perforator or two perforators for the flap was not associated with developing bulges ( *p* = 0.19) or hernias ( *p* = 0.47). The specific perforator row used was not associated with developing bulges for left ( *p* = 0.95) or right ( *p* = 0.83) sided flaps or hernias for left ( *p* = 0.40) or right ( *p* = 0.80) sided flaps. There were no differences in other abdominal wall complications between the cohorts, including dehiscence ( *p* = 1.0), SSI ( *p* = 1.0), seroma ( *p* = 0.77), or hematoma formation ( *p* = 0.08). Additionally, there were no significant differences in ED visits related to abdominal symptoms ( *p* = 0.76) or reoperations for abdominal complications ( *p* = 1.0) between the cohorts ( Table 2 ).

**Table 2:** Postoperative abdominal-related complications

Postoperative abdominal outcomes	OviTex mesh, *N* (%)	No mesh, *N* (%)	*p* -Value
Seroma	6 (7.1)	6 (5.3)	0.77
Hematoma	3 (3.5)	0 (0)	0.08
Surgical site infection	3 (3.5)	4 (3.5)	1.0
Wound dehiscence	6 (7.1)	8 (7.0)	1.0
Bulge	0 (0)	6 (5.3)	0.04
Hernia	0 (0)	2 (1.8)	0.51
Related ED visits	4 (4.7)	7 (6.1)	0.76
Reoperations	2 (2.4)	3 (2.6)	1.0

Abbreviation: ED, emergency department.

Patient factors including age (OR 1.03, confidence interval [CI] 0.95–1.12, *p* = 0.46), BMI greater than 30 kg/m ^2^ (OR 2.12, CI 0.32–13.91, *p* = 0.43), as well as chronic lung disease including asthma and chronic obstructive pulmonary disease (OR 1.07, CI 0.15–7.43, *p* = 0.95) were not statistically associated with developing a postoperative bulge or hernia ( Table 3 ). Diabetes was associated with patients developing a postoperative bulge (OR 7.46, CI 1.06–52.55, *p* = 0.04). Diabetes was also associated with an increased risk of dehiscence of the abdominal incision ( *p* = 0.04). No other risk factors were associated with wound dehiscence. In addition, there was not a statistically significant association between operative characteristics and bulge development including the surgeon performing the procedure (OR 0.74, CI 0.29–1.88, *p* = 0.53), unilateral or bilateral DIEP flap reconstruction (OR 0.81, CI 0.13–4.96, *p* = 0.82), and timing of DIEP flap reconstruction (OR 0.93, CI 0.14–6.30, *p* = 0.94).

**Table 3:** Multiple logistic regression analysis of preoperative and operative characteristics and their association with postoperative bulge development

Preoperative and operative characteristics	Odds ratio	Lower 95% CI	Upper 95% CI	*p* -Value
Age	1.03	0.95	1.12	0.46
BMI >30 kg/m ^2^	2.12	0.32	13.91	0.43
Chronic lung disease	1.07	0.15	7.43	0.95
Diabetes	7.46	1.06	52.55	0.04
Surgeon	0.74	0.29	1.88	0.53
Unilateral or Bilateral DIEP flap	0.81	0.13	4.96	0.82
Immediate or Delayed DIEP flap	0.93	0.14	6.30	0.94

Abbreviations: BMI, body mass index; CI, confidence interval; DIEP, deep inferior epigastric perforator.

## Discussion

Abdominal bulges and hernias are known complications following DIEP flap harvest. Existing literature suggests that the use of synthetic meshes to reinforce the abdominal wall fascia can improve the rate of bulges, but can be associated with several other complications making it not an ideal option. 1 2 3 11 Our study highlights the efficacy of using OviTex mesh for abdominal wall reinforcement in the setting of DIEP flap harvest for autologous breast reconstruction. In fact, our data demonstrated that the use of OviTex mesh significantly reduced the rate of bulges from 5.3% to 0%. However, there was a small number of patients (two, 1.8%) who developed hernias in our study, which did not allow us to make a meaningful comparison between the mesh and non-mesh groups. Our results are consistent with limited prior research evaluating the use of semiabsorbable mesh for reinforcement of the abdominal wall fascia following DIEP flap harvest that demonstrated reduction in the rate of bulges from 5.5% to 0% and hernias from 13.5% to 2.8% with the use of mesh. 12 19

One of the major complications associated with the use of synthetic mesh for reinforcement of the abdominal wall fascia following DIEP flap harvest is increased rates of seroma formation. It is not surprising that this complication is more common when the mesh is used in an onlay rather than a sublay fashion. 13 20 This was also noted when biologic meshes were used for this patient population. 21 22 23 Moreover, in a study by Rhemtulla et al., rates of SSI and reoperation were increased in patients who underwent abdominal wall fascia reinforcement with a biologic mesh following DIEP flap harvest. 21 This is in contrast to our study, which did not find a significant difference in rates of seroma formation, SSIs, or reoperations with the use of OviTex mesh. It is also important to mention that in our study drains were placed in the space above the anterior rectus sheath and not in the space where the mesh was placed. Our positive findings could be related to a variety of reasons including the permeability of OviTex mesh, and the fact that the mesh was placed in the retro-rectus space that may potentially allow faster and easier incorporation.

Acellular dermal matrices (ADMs) are another subset of biological meshes composed of a decellularized extracellular matrix collected as a full-thickness section of skin from a donor source (human, bovine, or porcine). 24 OviTex mesh that was used in this study is derived from ovine (sheep) rumen. 15 Unlike OviTex mesh, ADMs do not consist of multiple layers reinforced by either a resorbable or permanent suture. ADMs have been previously evaluated for use in reinforcing the abdominal wall fascia following DIEP flap harvest to help prevent bulges and hernias. 3 In a study by Haddock et al., 644 patients were evaluated for abdominal weakness, hernia, and bulge following DIEP flap harvest. 3 Of these, 38 patients had an ADM underlay placed in the initial abdominal wall repair, superficial to the rectus muscle and deep to the fascia. 3 It was found that the ADM cohort and non-ADM cohort did not significantly differ in the rate of postoperative bulges and hernias (3% vs. 5%, *p* = 0.64). 3 Additionally, a prior meta-analysis evaluating a human ADM used for any abdominal wall reconstruction, found a significant increase in the rate of bulges and hernias with its use. 25 In contrast, our study found that the use of OviTex mesh significantly decreased the rate of bulges following DIEP flap harvest. This suggests that the reinforcement provided by OviTex mesh may allow for enhanced tissue integration which aids in the strengthening of the abdominal wall thus minimizing the risk for postoperative bulges. In addition, OviTex mesh provides a cost advantage over some other absorbable meshes available at our institution. At our institution, the cost of OviTex for a 10 cm × 20 cm mesh is $1,800.00, which is a little over 40% cheaper when compared to other available absorbable biosynthetic meshes of similar size. Even though a fully synthetic polypropylene mesh of 10 cm × 14 cm is cheaper with a cost of $238.00, it is a permanent mesh and has been associated with increased complications. 13 14 15 In addition, when the various mesh options are presented to patients, many of our patients do not desire to have a permanent mesh. Further research on the use of OviTex for abdominal wall reinforcement in the setting of DIEP flap harvest is needed to directly compare its efficacy to other available biological and/or bioabsorbable meshes.

Risk factors for the development of bulges and hernias have been previously evaluated in an attempt to determine when abdominal wall reinforcement should be utilized. 2 26 27 28 However, there has been debate over which factors influence outcomes the most. For example, Butler et al. and Rezania et al. found that patients with higher BMIs were more likely to develop a postoperative bulge or hernia, whereas Jakeman et al. did not find such an association with BMI. 2 11 28 Additionally, Huang et al. demonstrated that BMI was associated with increased donor site complication. 29 Initially, the authors selectively chose to use OviTex in patients with higher BMIs but as they noticed decreased bulge rates they implemented OviTex mesh in all patients regardless of risk factor. Our study also did not find a statistically significant association between a BMI greater than 30 kg/m ^2^ and postoperative bulge development (OR 2.12, CI 0.32–13.91, *p* = 0.43). Another study by Nahabedian and Momen showed no association between diabetes and tobacco use with the development of a postoperative bulge or hernia in patients undergoing unilateral or bilateral DIEP flap harvest. 26 However, our study found that there was an association between patients with diabetes and developing a bulge (OR 7.46, CI 1.06–52.55, *p* = 0.04). This may be due to decreased collagen density and contraction during wound healing in patients with diabetes. 30 Additionally, a study by Wen et al. demonstrated that an increased operative time has been significantly associated with increased bulges postoperatively with an increase of 25% per hour of operative time. 31 Importantly, our study demonstrated that there was no significant increase in operative time with mesh placement ( *p* = 0.65).

Perforator type and number of perforators have previously been evaluated as risk factors associated with abdominal donor site morbidity including bulges and hernias after DIEP flap harvest. Several prior studies demonstrated that using both medial and lateral perforators or two rows of perforators results in a higher incidence of postoperative bulges or hernias. 2 32 This may be due to a greater degree of dissection required when multiple perforators are used. Elver et al. found that the use of both medial and lateral rows of perforators was associated with a higher incidence of hernia or bulge compared to using a single row, although mesh was more commonly used in both row flaps to mitigate this risk. 32 Butler et al. also noted that harvesting two or more perforators, especially from the lateral row or both rows, was more likely to result in postoperative abdominal hernia or bulge. 2 Several studies have investigated the impact of perforator type on abdominal donor site morbidity. Garvey et al. found no significant difference in abdominal bulge or hernia rates between medial and lateral branch perforator harvests, suggesting that the choice of perforators should be based on quality, size, and orientation rather than location to reduce donor site morbidity. 33 However, Hembd et al. reported that using lateral row or both medial and lateral row perforators increased the odds of abdominal bulge or hernia compared to medial row perforators (OR 3.21, *p* = 0.05). 34 Similarly, Grünherz et al. identified a significant correlation between the use of lateral row perforators and an increased risk of abdominal bulging ( *p* = 0.009). 35 Our study did not find an association with bulges or hernias and the type of perforator used for left ( *p* = 0.95, *p* = 0.40, respectively) or right ( *p* = 0.83, *p* = 0.80, respectively) sided flaps or the number of perforators harvested (bulges [ *p* = 0.19] or hernias [ *p* = 0.47]). Our findings align with a prior study by Garvey et al. that did not find differences in perforator type and rates of bulges and hernias. 33 This suggests that other factors such as surgical technique and the use of mesh may mitigate the risk of developing a bulge or hernia.

Our study should be reviewed within the context of some limitations. This was a single-institution study that evaluated patients from multiple surgeons in a retrospective nature, and may not be generalizable to other institutions. Despite the use of different suture materials for the primary fascial closure among the participating surgeons, the surgical technique for mesh placement was the same and our results showed that the surgeon performing the surgery was not associated with bulge development (OR 0.74, CI 0.29–1.88, *p* = 0.53). A multi-institutional study with a standardized approach to the entire abdominal wall closure can potentially address some of these limitations in the future. Another limitation is that we did not measure the fascial cut for each patient, and although all the surgeons preserved as many nerves as possible, we did not count or always reconnect all the nerves during lateral row harvest. Additionally, bulges could be underreported in both groups, especially in patients with a higher BMI. This study was also limited by the follow-up period. Due to the recent implementation of the use of OviTex meshes at our institution, the patients in the OviTex cohort had a shorter follow-up period compared to the non-mesh cohort. While this does not limit the findings of acute postoperative complications, it may underestimate the overall rate of late postoperative bulges and hernias considering that surgical complications can occur years after a procedure. Despite that, we utilized a minimum of 12-month follow-up—a time interval that allows for the development of most abdominal wall-related complications. 36 We plan to continue to monitor these patients and provide future updates with longer follow-up periods.

## Conclusion

This study sought to evaluate the efficacy and safety of OviTex mesh for retro-rectus abdominal wall reinforcement following DIEP flap harvest. Our results demonstrated that OviTex mesh is not associated with increased rates of seroma, SSI, or wound dehiscence when compared to no mesh use, and it is efficacious at minimizing the risk of abdominal bulges. Bulges are difficult complications to address postoperatively, therefore, plastic surgeons should consider using OviTex during DIEP flap harvest to prevent bulges, especially in patients with diabetes. Although our sample size is modest, the results of this study are clinically significant and very relevant to surgeons using the DIEP free flap for reconstructive or cosmetic purposes. Our findings highlight the need for continued investigation to assess the efficacy of OviTex mesh and other meshes in minimizing postoperative complications for this patient population. A prospective multicentered study comparing various meshes with an extended follow-up period is necessary to further enhance our understanding of the utility of meshes for reinforcement of the abdominal wall following DIEP flap harvest.
